# Multi-pathway mechanisms of Liujunzi decoction in promoting glioma apoptosis and reversing drug resistance: network pharmacology and experimental validation

**DOI:** 10.3389/fphar.2025.1682550

**Published:** 2025-09-24

**Authors:** Jinxiang Huang, Shengnan Lin, Lei Lin, Luning Xu, Dezhi Kang

**Affiliations:** ^1^ Department of Neurosurgery, Neurosurgery Research Institute, The First Affiliated Hospital of Fujian Medical University, Fuzhou, China; ^2^ Department of Neurosurgery, National Regional Medical Center, Binhai Campus of the First Affiliated Hospital of Fujian Medical University, Fuzhou, China; ^3^ Department of Clinical Pharmacy, Sanming First Hospital Affiliated to Fujian Medical University, Sanming, China; ^4^ Fujian Institute of Brain Disorders and Brain Science, The First Affiliated Hospital, Fujian Medical University, Fuzhou, China

**Keywords:** glioma, Liujunzi decoction, molecular docking, network pharmacology, drug resistance

## Abstract

**Background:**

Glioma is a highly aggressive brain tumor with a poor prognosis. Recent studies have demonstrated the anti-tumor potential of Liujunzi decoction (LJZD), but its specific effects and mechanisms in glioma remain unclear. This study aimed to elucidate the anti-glioma effects of LJZD and explore its underlying therapeutic mechanisms.

**Methods:**

The active ingredients of LJZD were retrieved from the TCMSP database and screened using ADME analysis. Potential targets of both the ingredients and glioma were retrieved from various databases. The overlapping targets were identified as LJZD’s therapeutic targets for glioma. To elucidate the biological functions and potential association among the overlapping targets, protein-protein interaction (PPI) network analysis, Gene Ontology (GO) and Kyoto Encyclopedia of Genes and Genomes (KEGG) enrichment analyses were conducted. Molecular docking was employed to evaluate the binding affinities between core targets and LJZD’s main active ingredients. The inhibitory effect of LJZD on U251 glioma cells was validated by *in vitro* experiments.

**Results:**

In total, we identified 76 active ingredients and 752 potential targets of LJZD, while 1,456 glioma-related targets were collected from databases, yielding 174 overlapping targets through intersection. PPI network analysis revealed 40 hub targets, with TP53, AKT1, HIF1A, TNF and IL6 ranking as the top five core targets based on degree. GO and KEGG enrichment analyses demonstrated that LJZD’s anti-glioma effects are mediated through genes related to apoptosis and key pathways including platinum drug resistance, EGFR tyrosine kinase inhibitor resistance, and PD-LI expression and PD-1 checkpoint pathway in cancer. Molecular docking confirmed good to strong binding affinities between LJZD’s main active ingredients (glabridin, pinocembrin and chrysophanol) and all top 10 core targets. *In vitro* experiments showed that glabridin inhibited U251 cell viability and promoted apoptosis. Glabridin significantly downregulated phosphorylation levels of AKT, STAT3 and c-Jun, while reducing expression of c-Myc and HIF-1α in U251 cells.

**Conclusion:**

LJZD may exert anti-glioma effects by inducing tumor cell apoptosis and overcoming drug resistance, potentially serving as an adjuvant to enhance conventional therapies. Additionally, its active ingredients (e.g., glabridin, pinocembrin and chrysophanol) provide novel leads for developing small-molecule therapeutics.

## 1 Introduction

Glioma is a common malignant intracranial tumor, accounting for 80% of all malignant intracranial tumors (Schaff and Mellinghoff, 2023). Current treatment options primarily include surgery, radiotherapy, and chemotherapy, with the addition of tumor-treating fields showing some survival benefit (Stupp et al., 2017). Even with combined surgery and postoperative radiochemotherapy, the median survival is only around 14 months (Stupp et al., 2005; Stupp et al., 2009). Although immunotherapy and targeted therapy have recently emerged as potential treatments for glioma, the overall prognosis for patients remains poor, with many tumors eventually recurring. Therefore, the development of more effective glioma treatment strategies remains a key area of research.

Traditional Chinese medicine (TCM) and formulas are a treasure of Chinese civilization, with a history of thousands of years of application in China. Liujunzi decoction (LJZD), derived from the “Yixue Zhengzhuan”, is composed of six Chinese herbal ingredients, which are *Panax Ginseng C. A. Mey.*, *Atractylodes Macrocephala Koidz*., *Poria Cocos (Schw.) Wolf.*, *licorice*, *Arum Ternatum Thunb.*, and *Citrus Reticulata*. It is based on the four ingredients of Sijunzi decoction with the addition of *Arum Ternatum Thunb.* and *Citrus Reticulata*. Therefore, in addition to its original functions of invigorating Qi and strengthening the spleen, LJZD also has the effects of drying dampness, resolving phlegm, regulating Qi and harmonizing the stomach. Historically, LJZD has been used to treat syndromes of spleen-stomach qi deficiency accompanied by phlegm-dampness or qi stagnation. Recent studies have revealed its potential antitumor effects. For instance, according to Guo et al. (2024), LJZD has the ability to enhance the expression of miR-122-3p in hepatocellular carcinoma cells. This action leads to the downregulation of UBE2I and the inhibition of the NF-κB/PD-L1 pathway, effectively suppressing SUMOylation in cancer cells and safeguarding PD-1^+^ T cells co-cultured with tumor cells from undergoing apoptosis. Additionally, it has been demonstrated that LJZD can suppress the growth of esophageal cancer cells while boosting antitumor immune responses through elevated IFN-γ levels and enhanced infiltration of CD8^+^ tumor-infiltrating lymphocytes (Han et al., 2023). Furthermore, LJZD has been reported to improve anorexia associated with tumors or chemotherapy (Wu et al., 2022; Sun et al., 2020), suggesting its potential as an adjuvant in cancer therapy. However, whether LJZD has similar antitumor effects on glioma remains unclear.

To further elucidate the potential role of LJZD in glioma treatment, we employed a network pharmacology approach to analyze the main active ingredients of LJZD and their targets. By intersecting these targets with glioma-related targets, we constructed a target network elucidating LJZD’s anti-glioma mechanism. Core targets identified from this network were subjected to molecular docking with LJZD’s active ingredients to evaluate binding stability based on binding energy. Finally, we evaluated the effects of LJZD’s key active ingredients on glioma cell biological behaviors (cell viability, clonogenicity and apoptosis) as well as the expression patterns of core targets and their downstream molecules *in vitro*.

## 2 Materials and methods

### 2.1 Screening active ingredients and targets of LJZD

Traditional Chinese Medicine Systems Pharmacology Database and Analysis Platform (TCMSP, version 2.3) (Ru et al., 2014) serves as a specialized systems pharmacology platform for Chinese herbal medicines, illuminating the complex relationships between drugs, their targets, and associated diseases, and it has become a cornerstone in TCM research. The classic LJZD formula consists of six herbs: *Panax Ginseng C. A. Mey.*, *Atractylodes Macrocephala Koidz*., *Poria Cocos (Schw.) Wolf.*, *licorice*, *Arum Ternatum Thunb.*, and *Citrus Reticulata*. We searched for the active ingredients of these six herbs from TCMSP. The SMILES of the active ingredients were input into the SwissADME website (Daina et al., 2017) for ADME pharmacokinetic analysis. Initially, we selected ingredients with “High” oral bioavailability and “Yes” blood-brain barrier permeability. Subsequently, we implemented a modified Lipinski-based screening strategy: while retaining the classic thresholds for molecular weight (≤500 Da), lipophilicity (XLOGP ≤5), hydrogen bond donors (≤5), and acceptors (≤10), we introduced an additional molecular weight cutoff (>200 Da) to exclude excessively small molecules with potentially insufficient target engagement. Additionally, ingredients that met two or more of the Extended Drug-Likeness Rules (Ghose, Veber, Egan, and Muegge) were defined as the active ingredients of LJZD. The targets of the active ingredients of LJZD were predicted using SwissTargetPrediction (Daina et al., 2019), Herb (version 2.0) (Gao et al., 2025), and BATMAN-TCM (version 2.0) (Kong et al., 2024) platforms. We incorporated all targets predicted by Herb, retained targets with probability >0.6 from SwissTargetPrediction, and selected both known targets and predicted targets with score >80 from BATMAN. The targets obtained from the three websites were combined and deduplicated to obtain the potential targets for LJZD.

### 2.2 Glioma-related targets screening

We searched for glioma-related targets in public databases using “glioma” as the keyword. The databases and screening criteria are as follows: In the GeneCards database (Stelzer et al., 2016), we included targets with a relevance score >5. In the Online Mendelian Inheritance in Man (OMIM) database (Amberger et al., 2015), all targets were included. In the DisGeNET database (Pinero et al., 2020), we included targets with scoreGDA >0.6. In the Open Targets Platform (Buniello et al., 2025), we included targets with globalscore >0.6. In the Comparative Toxicogenomics Database (Davis et al., 2025), we included targets with inference score >40. All targets were combined and deduplicated to form the set of glioma-related targets.

### 2.3 Construction of protein-protein interaction (PPI) network and bioinformatics analysis

To identify the potential targets of LJZD against glioma, we intersected the targets of LJZD with the glioma-related targets and constructed a Venn diagram (Bardou et al., 2014). The intersecting targets were input into the STRING database (Version 12.0) (www.string-db.org) for PPI analysis, with the species set as “*Homo sapiens*” and a medium confidence score of 0.4. The PPI network was visualized using Cytoscape 3.10.3. We analyzed the network using Centiscape 2.2 to obtain the betweenness, closeness, and degree values for each node, which reflect the importance of the nodes in the network. We calculated the average betweenness, closeness, and degree values for all intersecting targets and selected targets with values above the average as hub targets. The interaction network of hub targets was constructed using Cytoscape 3.10.3.

To further understand the functions of the targets related to LJZD’s treatment of glioma and the affected signaling pathways, we performed Gene Ontology (GO) and Kyoto Encyclopedia of Genes and Genomes (KEGG) pathway enrichment analyses on the intersecting targets using the DAVID bioinformatics analysis platform (Sherman et al., 2022; Huang da et al., 2009). The GO analysis covered three categories: biological process (BP), cellular component (CC), and molecular function (MF). For the KEGG pathway enrichment analysis, we used an FDR <0.05 as the cut-off value. The results of the GO and KEGG pathway enrichment analyses were visualized using the bioinformatics platform (www.bioinformatics.com.cn).

### 2.4 Molecular docking

To further elucidate the interactions between the active ingredients of LJZD and core targets, we constructed a network of intersection targets and active ingredients using Cytoscape 3.10.3. We selected the top 10 active ingredients with the highest degree as the main active ingredients for glioma treatment. Similarly, we identified the top 10 targets with the highest degree from the PPI network analysis as core targets. Subsequently, we performed molecular docking between the top 10 active ingredients and core targets to assess their binding affinities. The 3D structure files of the active ingredients were obtained from PubChem (Kim et al., 2023). The 3D crystal structures of the top 10 core target proteins were retrieved from the RCSB Protein Data Bank (rcsb.org) (Berman et al., 2000). Molecular docking was conducted using AutoDock Vina (Version 1.2.0) (Eberhardt et al., 2021). We recorded the binding energies of the active ingredients and target proteins and visualized these interactions using a binding energy heatmap. Lower binding energies indicate higher binding affinities. Generally, binding energies below −5.0 kcal/mol are considered good binding, while those below −7.0 kcal/mol are considered strong binding. The PDB files of the optimal binding conformations of the active ingredients and core targets were submitted to the Protein-Ligand Interaction Profiler website for intermolecular interaction analysis (Adasme et al., 2021). The results were visualized using PyMOL software (Version 3.11).

### 2.5 Experimental validation of anti-glioma effect of LJZD

#### 2.5.1 Chemicals and reagents

The active ingredients of LJZD, including pinocembrin (HY-N0575), glabridin (HY-N0393), and chrysophanol (HY-13595), were purchased from MedChemExpress (Monmouth Junction, NJ, USA). CCK-8 kit was obtained from Sangon Biotech (Shanghai, China; Cat. No. E606335-0500), while the apoptosis detection kit (Cat. No. A211-01) was sourced from Vazyme (Jiangsu, China). The antibodies used in the Western blot were purchased from HUABIO (Hangzhou, China), with the following details: AKT (ET1609-51, 1:1,000), phosphorylated AKT (p-AKT, ET1607-73, 1:1,000), phosphorylated STAT3 (p-STAT3, ET1603-40, 1:1,000), phosphorylated c-Jun (p-c-Jun, HA722475, 1:1,000), c-Myc (HA722895, 1:1,000), HIF-1α (HA721997, 1:1,000), Cleaved caspase 3 (HA722367, 1:1,000). Antibody for GAPDH was purchased from Cell Signaling Technology (#5174, CST, 1:1,000).

#### 2.5.2 Cell lines and culture

The human glioma U251 cell line was obtained from Shanghai Fuheng Biology (Shanghai, China). The cells were cultured in high-glucose Dulbecco’s Modified Eagle Medium supplemented with 10% fetal bovine serum and 1% penicillin/streptomycin at 37 °C with 5% CO_2_. Cell dissociation was performed using 0.25% trypsin (German-Cortes et al., 2025). Log-phase cells were used for subsequent experiments.

#### 2.5.3 Cell viability assay

Logarithmically growing cells were digested, washed and then resuspended in culture medium. After cell counting, the cells were seeded into 96-well plates at a density of 20,000 cells per well for the CCK8 assay. To determine the half-maximal inhibitory concentrations (IC_50_) of the selected ingredients from LJZD against glioma, U251 cells were treated with gradient concentrations. After 24 h of incubation, 10 μL of CCK8 reagent was added to each well, and the plates were further incubated at 37 °C in the dark for 40 min. Absorbance was measured at 450 nm. The IC_50_ values of the selected ingredients were calculated using GraphPad Prism software (Ge et al., 2024). Each concentration was tested in quintuplicate.

To evaluate the inhibitory effects of glabridin on the viability of U251 cells, low (40 μM), medium (80 μM), and high (120 μM) concentrations of glabridin were added to the wells based on its IC_50_ value, with an equivalent volume of DMSO serving as the control. After incubation for 24, 48, and 72 h, the culture medium was replaced, and 10 μL of CCK8 reagent was added to each well. Absorbance was measured at 450 nm following further incubation.

#### 2.5.4 Colony formation assay

U251 cells were seeded into 6-well plates at a density of 1,000 cells per well and cultured for 2 weeks. During this period, cells were treated with low (40 μM), medium (80 μM), and high (120 μM) concentrations of glabridin, with cells treated with DMSO serving as the control group. At the end of the culture period, cell colonies were fixed with paraformaldehyde and stained with crystal violet. The stained colonies were photographed and counted under a microscope to assess the impact of glabridin on cell colony-forming ability.

#### 2.5.5 Apoptosis assay

To assess apoptosis, U251 cells were plated in 6-well plates. The cells were then exposed to varying concentrations of glabridin—low (40 μM), medium (80 μM), and high (120 μM)—with DMSO used as a control. Following 24 h of treatment, the cells were gathered, centrifuged to discard the supernatant, and resuspended in binding buffer. Annexin V-PE-CY7 and propidium iodide were introduced and mixed gently. After a 15-min incubation at room temperature in the dark, the cells were subjected to flow cytometry analysis. Each experiment was conducted in triplicate.

#### 2.5.6 Reverse transcription polymerase chain reaction (RT-PCR)

U251 cells were exposed to 80 μM glabridin. Following a 24-h incubation period, total RNA was isolated using the TRIzol reagent kit (Invitrogen; Carlsbad, CA, USA). The RNA concentration was quantified via spectrophotometry. Subsequently, the RNA was reverse transcribed into cDNA. For the amplification process, specific primers for the target genes (Supplementary Table S1) were incorporated into the cDNA samples. The amplification protocol involved an initial denaturation at 95 °C for 2 min, followed by 40 cycles consisting of 15 s at 95 °C and 30 s at 60 °C ((Ma et al., 2025)). GAPDH was used as an internal control (Tang et al., 2024). The mRNA expression levels were normalized to GAPDH using the 2^−ΔΔCt^ method.

#### 2.5.7 Western blotting

U251 cells were treated with either glabridin (80 μM) or DMSO for 24 h. After washing the cells with PBS, lysis buffer was added, and the cells were incubated before centrifugation to obtain total protein. Protein concentration was measured using a BCA protein assay kit. The protein samples were mixed with 5× SDS-PAGE loading buffer and boiled at 100 °C for 5 min to denature the proteins. The proteins were then separated on a 10% SDS-PAGE gel and transferred to a PVDF membrane. The membrane was blocked with 5% non-fat milk and incubated overnight at 4 °C with primary antibodies, using GAPDH as an internal control. After washing to remove excess primary antibodies, the membrane was incubated with secondary antibodies at 37 °C. Chemiluminescence was detected using an ECL kit. The gray values of the Western blot bands were analyzed using ImageJ software.

### 2.6 Statistical analysis

All data are presented as the mean ± standard deviation. Statistical analyses were conducted using GraphPad Prism 10 (GraphPad Software, Boston, MA, USA). Comparisons between two groups were performed using the t-test, while comparisons among multiple groups were analyzed using one-way analysis of variance (ANOVA), followed by Sidak’s test for post-hoc pairwise comparisons. A *P*-value of less than 0.05 was considered statistically significant.

## 3 Results

### 3.1 Targets associated with LJZD’s anti-glioma activity

**FIGURE 1:**
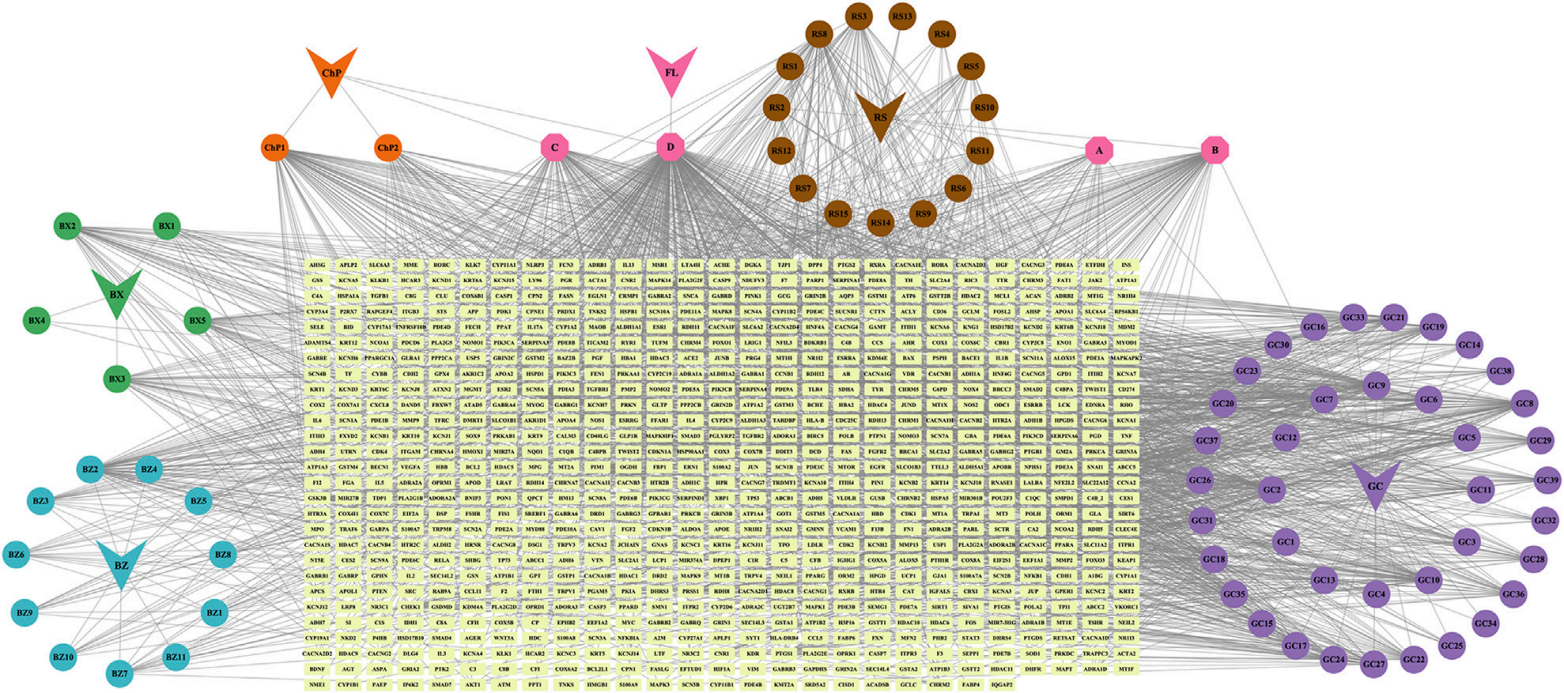
Active ingredients and targets of Liujunzi decoction (LJZD). The herb-ingredient-target network of LJZD, where the V-shapes represent the six herbs in LJZD, the circles connected to the V-shapes represent the active ingredients derived from the herbs, and the magenta hexagons **(A–D)** indicate active ingredients shared by two or more herbs. The light-colored square blocks in the center represent all targets corresponding to the active ingredients. RS (Ren Shen): Panax ginseng **(C) (A)**. Mey., BZ (Bai Zhu): Atractylodes macrocephala Koidz., FL (Fu Ling): Poria cocos (Schw.) Wolf., GC (Gan Cao): licorice, BX (Ban Xia): Pinellia ternata (Thunb.), ChP (Chen Pi): Citrus reticulata Blanco. The names of the active ingredients represented by circles and hexagons are provided in Supplementary Table S1.

Using the TCMSP database, we identified 190 active ingredients in *Panax Ginseng C. A. Mey.*, 55 in *Atractylodes Macrocephala Koidz.*, 34 in *Poria Cocos (Schw.) Wolf.*, 63 in *Citrus Reticulata.*, 280 in *licorice*, and 116 in *Arum Ternatum Thunb.* We then analyzed the pharmacokinetic ADME properties of each active ingredient using SwissADME and selected 90 ingredients that met our criteria. Subsequently, we predicted the targets of these active ingredients using SwissTargetPrediction, Herb, and BATMAN, excluding 14 ingredients with no predicted targets. In total, 76 active ingredients and 752 targets were identified for LJZD (Figure 1; Supplementary Table S2).

**FIGURE 2:**
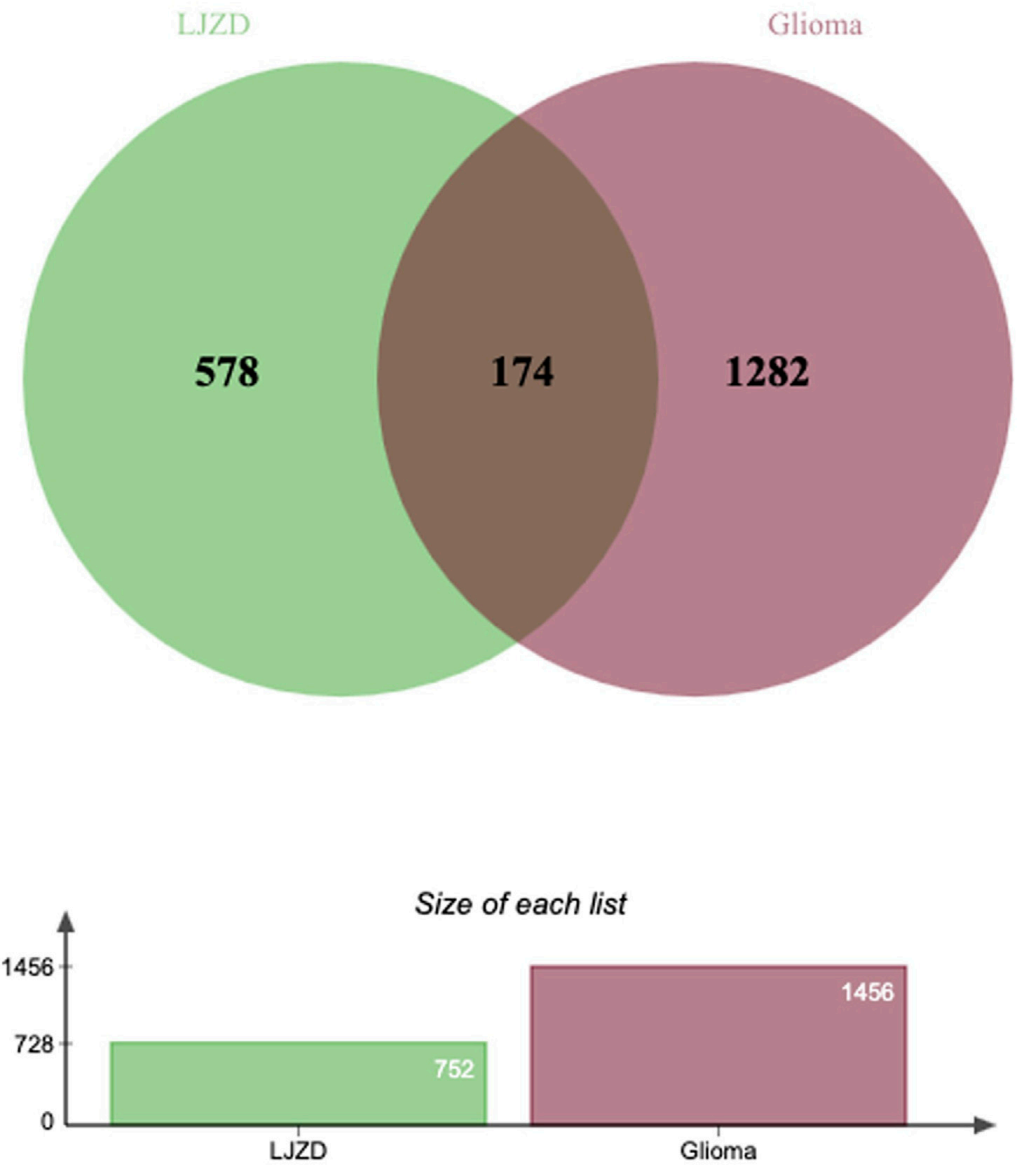
Venn diagram of targets from LJZD and glioma Green circles represent LJZD targets, dark red circles indicate glioma-related targets, and the overlapping areas between circles denote shared targets between LJZD and glioma.

**FIGURE 3:**
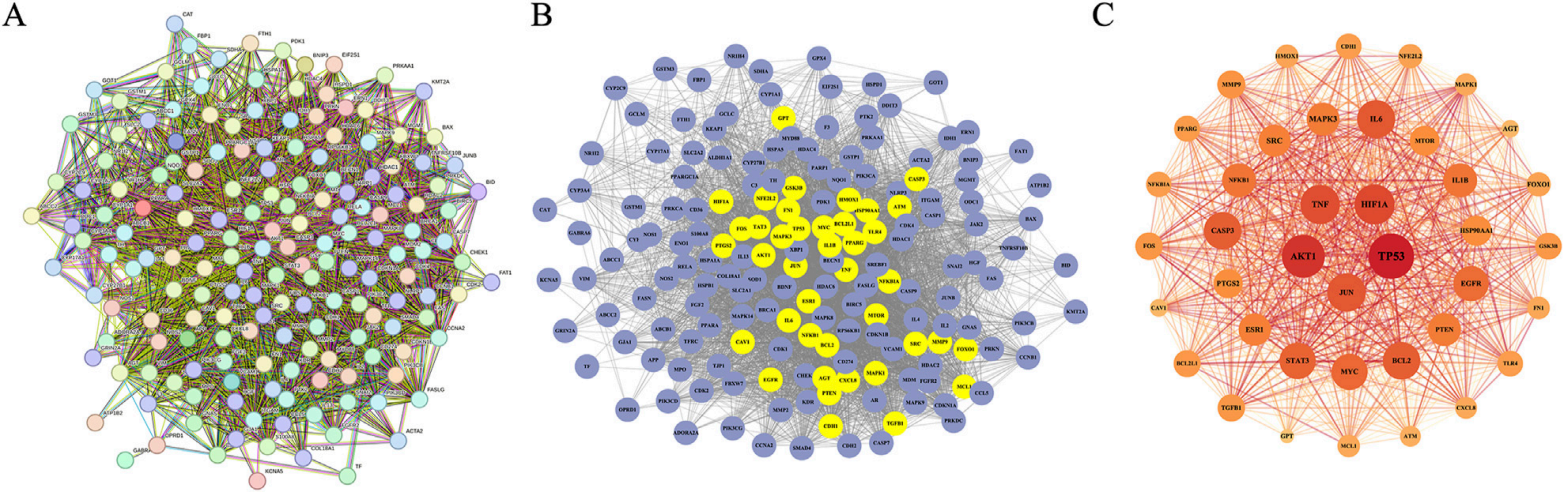
Protein-protein interaction (PPI) network of overlapping targets and core targets **(A)** PPI network of intersection targets between LJZD and glioma, constructed using the STRING database. **(B)** Screening process of hub targets from the intersection targets through PPI network analysis. Each circular node represents one target, with yellow nodes indicating the screened hub targets. **(C)** PPI network of hub targets, containing 40 nodes and 746 edges where each target is represented by a circle. The node size corresponds to degree with larger diameter indicating higher degree. The font size reflects betweenness where larger text indicates higher betweenness. The node color represents closeness with darker red indicating higher closeness. The connecting lines show interactions between targets where darker red and thicker lines indicate higher combined scores.

To identify glioma-related targets, we searched public databases including GeneCards, OMIM, DisGeNET, Open Targets, and CTD. Applying the screening criteria outlined in Section 2.2, we retrieved 1,022 targets from GeneCards, 196 from OMIM, 138 from DisGeNET, 41 from Open Targets, and 431 from CTD. After merging, we identified 1,456 glioma-related targets. Finally, we intersected the LJZD targets with the glioma-related targets, resulting in 174 intersection targets (Figure 2). To elucidate the relationships among these intersection targets, we constructed a PPI network using the STRING database (Figure 3A). This network comprises 172 nodes and 4932 edges, with mean betweenness, closeness, and degree values of 120.105, 0.0035, and 57.349, respectively. Among the 174 targets, 40 targets had betweenness, closeness, and degree values above the mean (Figure 3B) and were designated as hub targets. TP53 exhibited the highest betweenness, closeness, and degree, followed by AKT1, HIF1A, and TNF. Additionally, JUN, IL6, STAT3, and MYC ranked in the top 10, highlighting their significance in the hub network. The PPI network of hub targets is shown in Figure 3C.

### 3.2 GO and KEGG analysis of intersection targets

**FIGURE 4:**
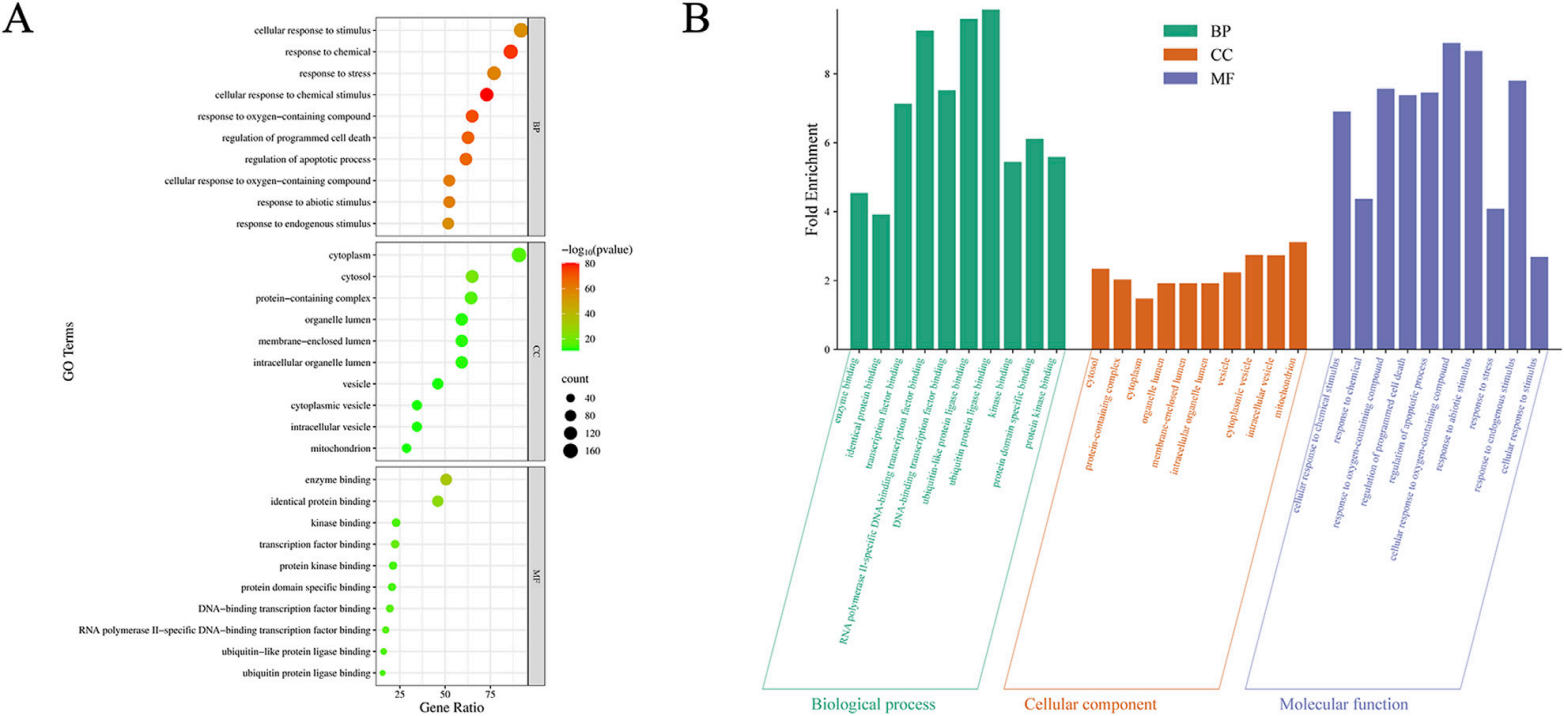
Gene Ontology (GO) analysis of intersection targets between LJZD and glioma. Bubble chart **(A)** and histogram **(B)** of GO analysis for intersection targets between LJZD and glioma, showing the top 10 terms in biological process (BP), cellular component (CC), and molecular function (MF) categories.

To further reveal the intracellular biological functions of intersection targets and their involvement in signaling pathways during LJZD treatment of glioma, we performed GO and KEGG analyses on the 174 intersection targets using the DAVID platform. The GO analysis yielded a total of 2556 significant terms (FDR <0.05), comprising 2164 BP terms, 243 MF terms, and 149 CC terms. Using the bioinformatics platform, we visualized the top 10 terms in each category (Figures 4A,B; Supplementary Table S3). As shown in Figure 4, LJZD’s effects on glioma primarily involve several intracellular biological processes, including enzyme binding, identical protein binding, transcription factor binding, ubiquitin protein ligase binding, protein domain-specific binding, and protein kinase binding. These findings highlight the potential of LJZD to regulate protein function and signal transduction mechanisms. The main intracellular ingredients implicated include the cytoplasm, cytosol, protein-containing complex, organelle lumen, and membrane-enclosed lumen. Molecular functions primarily involve cellular responses to stimuli (e.g., cellular response to chemical stimulus, response to chemical, response to oxygen-containing compound) and regulation of cell death (e.g., regulation of programmed cell death and regulation of apoptotic process).

**FIGURE 5:**
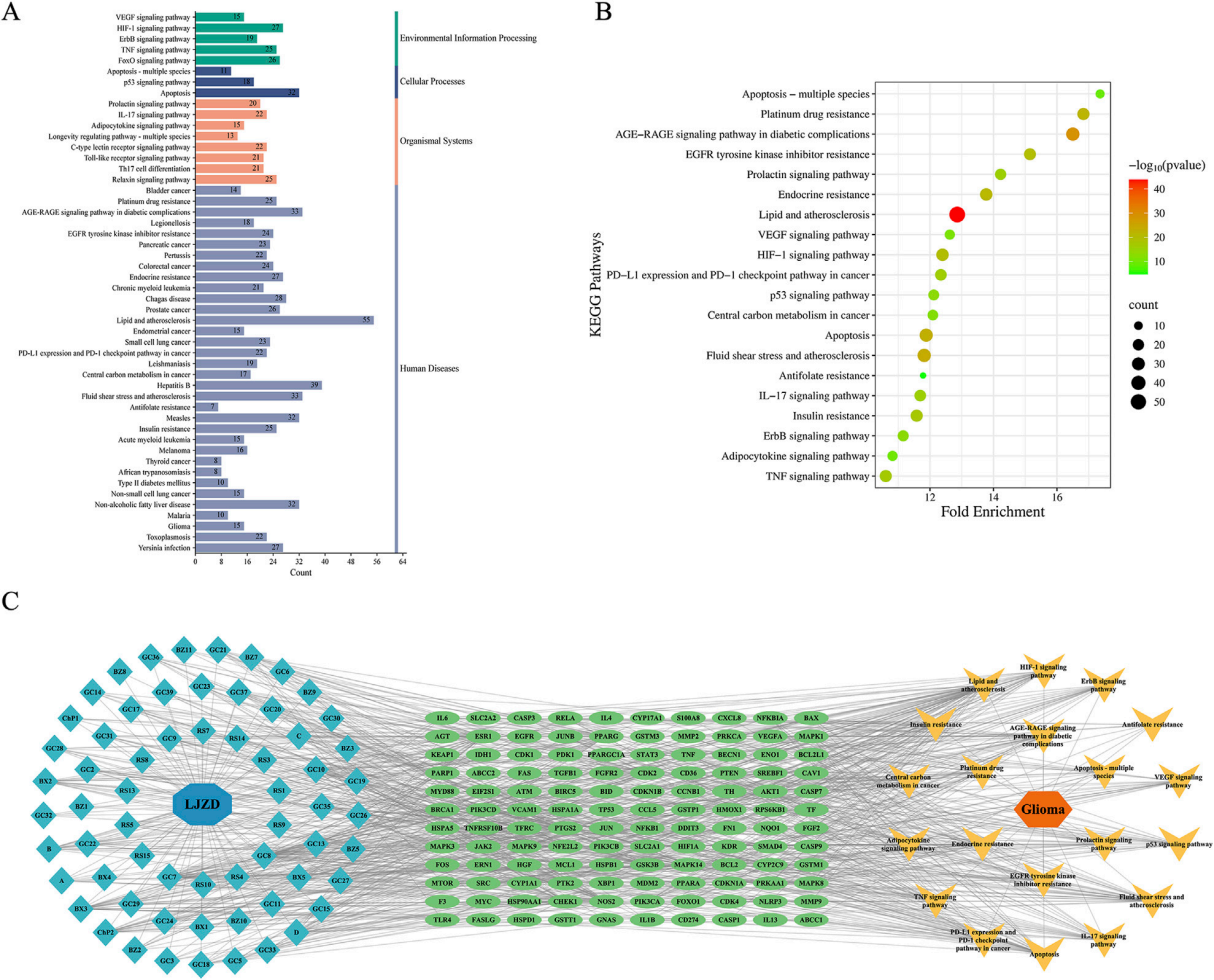
KEGG pathway enrichment analysis of intersection targets between LJZD and glioma. **(A)** Classification of KEGG pathway. The top 50 enriched KEGG pathways were categorized into four groups: Environmental Information Processing (green), Cellular Processes (dark blue), Organismal Systems (orange), and Human Diseases (gray). Numbers on the bars indicate the count of targets in each enriched pathway. **(B)** Bubble plot of the top 20 enriched KEGG pathways after removing non-glioma related tumors and specific disease entries from the Human Diseases category. **(C)** Formula-ingredient-target-pathway-disease network diagram. Yellow V-shaped nodes represent the top 20 KEGG pathways, green ellipse nodes denote their corresponding targets, and blue diamond nodes indicate active ingredients from LJZD.

KEGG pathway enrichment analysis identified a total of 170 pathways. As shown in Figure 5A, the top 50 pathways with the highest fold enrichment scores (Supplementary Table S4) include apoptosis and the p53 signaling pathway in cellular processes, suggesting that LJZD may induce apoptosis in glioma cells. Pathways in environmental information processing involve VEGF, HIF-1, ErbB, TNF, and FoxO signaling pathways. Additionally, several pathways are associated with human diseases, including various common cancers such as non-small cell lung cancer, colorectal cancer, and acute myeloid leukemia. To more clearly illustrate the specific molecular pathways affected by LJZD in glioma, we removed pathways related to other tumors or specific diseases and presented the top 20 pathways in a bubble chart (Figure 5B). Among the top 20 enriched pathways, those related to current cancer drug treatments, such as platinum drug resistance, EGFR tyrosine kinase inhibitor resistance, PD-L1 expression and PD-1 checkpoint pathway in cancer, and antifolate resistance, have high enrichment scores, indicating the potential of LJZD as a therapeutic agent for glioma. Based on these findings, we constructed a LJZD ingredient-target-pathway network (Figure 5C).

### 3.3 Molecular docking of core targets and major active ingredients of LJZD

**FIGURE 6:**
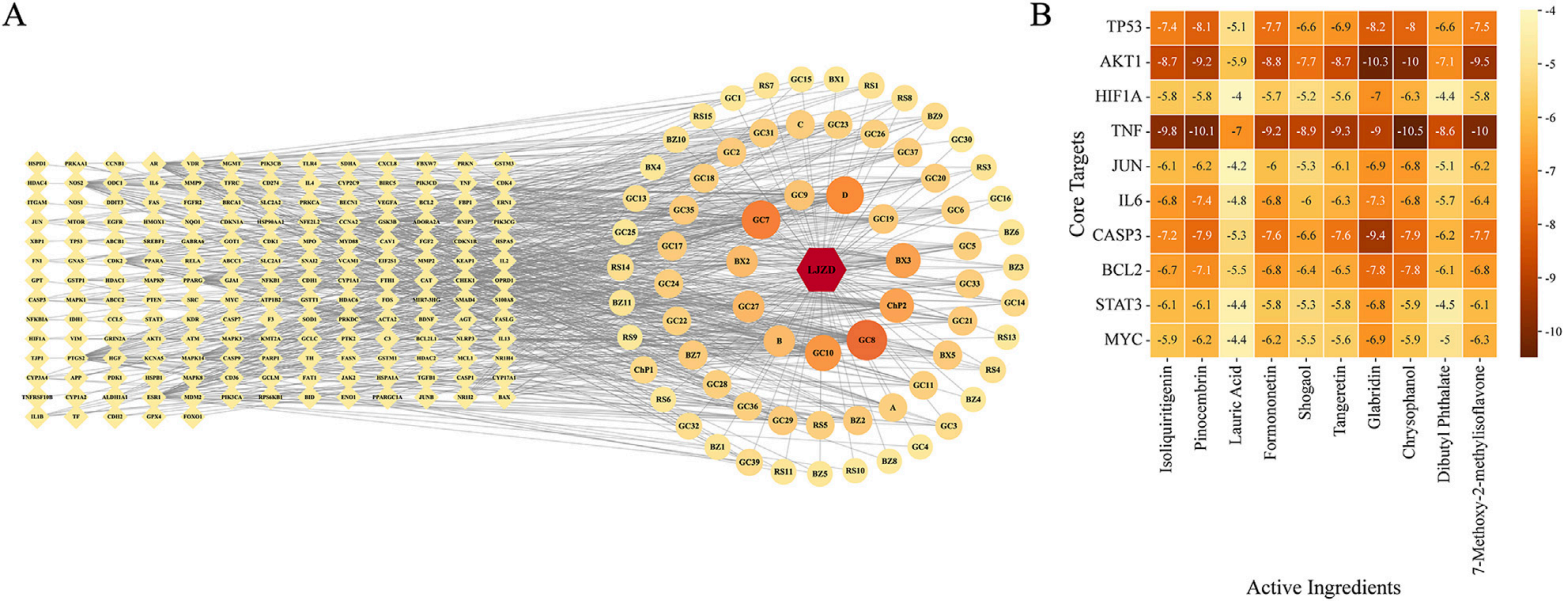
Screening of main active ingredients and molecular docking results of active ingredients and core targets. **(A)** Screening of key active ingredients. The light-yellow diamonds on the left represent the overlapping targets between LJZD and glioma, while the circles on the right represent the active ingredients. The size and color of the circles indicate their degree values—larger diameters and darker colors correspond to higher degree values. **(B)** Heatmap of molecular docking binding energies between the top 10 key active ingredients and the top 10 core targets. The numerical value in each square represents the binding energy, where lower values indicate higher affinity between the active ingredient and the target. The color intensity of the squares reflects affinity strength, with darker colors denoting higher affinity.

To identify the major active ingredients of LJZD that contribute to its anti-glioma effects, we constructed an intersection target-ingredient-formula network (Figure 6A). Network analysis revealed that the top five active ingredients (ranked by their degree) are isoliquiritigenin (degree = 44), pinocembrin (degree = 39), lauric acid (degree = 36), formononetin (degree = 32), and shogaol (degree = 28). In the interaction network, a higher degree indicates more connections with other nodes. In the intersection target-ingredient-formula network, the degree of active ingredients indicates their influence on the intersection targets. Based on this, we selected the top 10 active ingredients with the highest degree as the major active ingredients of LJZD for glioma treatment. Similarly, we identified the top 10 targets with the highest degree from the PPI network of intersection targets as core targets. To further elucidate the interactions between these major active ingredients and core targets (Tables 1, 2), we performed molecular docking analysis to assess their binding affinities and binding modes.

**TABLE 1:** The top 10 main active ingredients of LJZD.

Rank	Ingredient code	Name	PubChem CID	SMILES	Degree
1	GC8	Isoliquiritigenin	638278	C1 = CC(=CC = C1/C=C/C (=O)C2 = C(C=C(C=C2)O)O)O	44
2	GC7	Pinocembrin	68071	C1 [C@H](OC2 = CC(=CC(=C2C1 = O)O)O)C3 = CC = CC = C3	39
3	D	Lauric acid	3893	CCCCCCCCCCCC(=O)O	36
4	GC10	Formononetin	5280378	COC1 = CC = C(C=C1)C2 = COC3 = C(C2 = O)C=CC(=C3)O	32
5	BX3	Shogaol	5281794	CCCCC/C=C/C (=O)CCC1 = CC(=C(C=C1)O)OC	28
6	ChP2	Tangeretin	68077	COC1 = CC = C(C=C1)C2 = CC(=O)C3 = C(O2)C (=C(C(=C3OC)OC)OC)OC	27
7	GC27	Glabridin	124052	CC1(C=CC2 = C(O1)C=CC3 = C2OC [C@H](C3)C4 = C(C=C(C=C4)O)O)C	20
8	BX2	Chrysophanol	10208	CC1 = CC2 = C(C(=C1)O)C (=O)C3 = C(C2 = O)C=CC = C3O	19
9	B	Dibutyl Phthalate	3026	CCCCOC(=O)C1 = CC = CC = C1C(=O)OCCCC	19
10	GC9	7-Methoxy-2-methyl isoflavone	354368	CC1 = C(C(=O)C2 = C(O1)C=C(C=C2)OC)C3 = CC = CC = C3	16

**TABLE 2:** The top 10 core targets between LJZD and glioma.

Rank	Core protein	PDB ID	Degree
1	TP53	5O1C	151
2	AKT1	3O96	144
3	HIF1A	4H6J	136
4	TNF	6OOY	134
5	JUN	5FV8	131
6	IL6	1P9M	130
7	CASP3	1NM	128
8	BCL2	8HOG	128
9	STAT3	6NJS	125
10	MYC	1NKP	124

**FIGURE 7:**
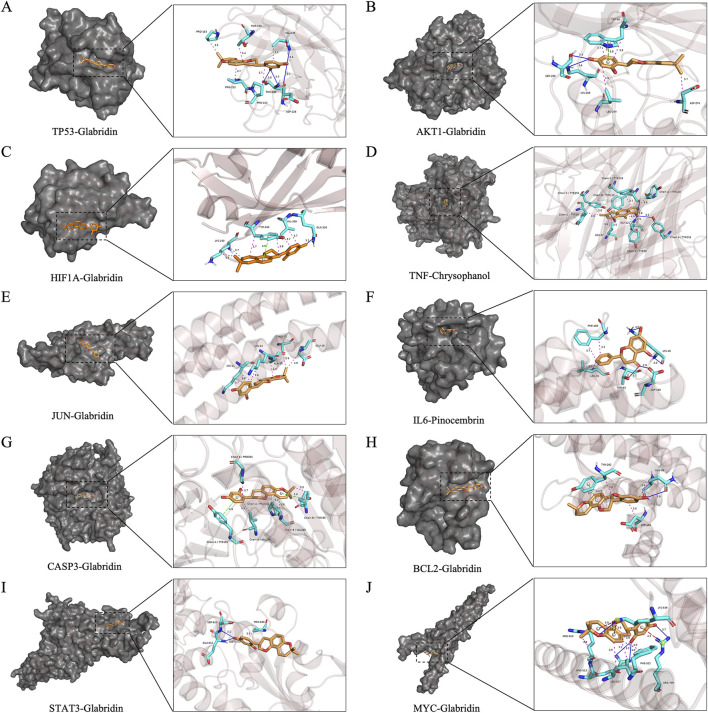
Binding conformations and molecular interactions between the top 10 core targets and their highest-affinity active ingredients. In this panel, the top 10 core targets are arranged from **(A–J)** in descending order of degree value, showcasing the binding conformations of these targets with their highest-affinity active ingredients. Each panel displays the binding conformation of the target protein with their highest-affinity active ingredient on the left, while the right side provides a magnified view of their molecular interactions. The protein structure is represented as a gray surface showing the binding pocket, with the active ingredient displayed in orange. Interacting amino acid residues are shown in light blue. Molecular interactions are indicated as follows: magenta dashed lines for hydrophobic interactions, dark blue solid lines for hydrogen bonds, green dashed lines for π-stacking, and red dashed lines for π-cation interactions. Bond lengths are indicated by numerical values adjacent to the interaction lines.

As shown in Figure 6B, except for lauric acid and dibutyl phthalate, the remaining eight active ingredients exhibited strong binding affinities (binding energy < −5 kcal/mol) with all 10 core proteins. For core targets such as TP53 (degree = 151), AKT1 (degree = 144), and TNF (degree = 134), most active ingredients demonstrated very strong binding (binding energy < −7 kcal/mol), highlighting their significant roles in LJZD’s anti-glioma effects. Among all 10 active ingredients, glabridin showed the highest binding affinity with eight out of 10 core targets (TP53, AKT1, HIF1A, JUN, CASP3, BCL2, STAT3, MYC). Chrysophanol exhibited the lowest binding energy with TNF (BE = −10.5 kcal/mol), while pinocembrin had the strongest binding affinity with IL6 (BE = −7.4 kcal/mol). We visualized the binding conformations and interactions of each core target-active ingredient pair with the lowest binding energy (Figure 7).

As illustrated in Figure 7, the hydroxyl group at the 4′-position of glabridin participates in the formation of hydrogen bonds within the binding conformations of multiple core proteins. This hydroxyl group acts as a hydrogen bond donor, forming hydrogen bonds with GLN320 in HIF-1α, GLN99 in BCL2, and ASP228 in TP53. It also acts as a hydrogen bond acceptor, forming hydrogen bonds with GLU612 and SER613 in STAT3, VAL147 in TP53, LYS268 and SER205 in AKT1, and ARG739 in MYC. In addition to these interactions, glabridin forms parallel π-stacking interactions with TYR340 in HIF-1α, TYR202 in BCL2, and TRP80 in AKT1. It also engages in π-cation interactions with LYS539 and ARG739 in MYC. In the binding conformation of pinocembrin with IL6, pinocembrin forms four hydrogen bonds with LYS46, ASP160, and THR43 of IL6. Additionally, it forms hydrophobic interactions with PHE105 and LEU39 of IL6, which together constitute the structural basis for the stable binding of pinocembrin to IL6 (binding energy = −7.4 kcal/mol). In the binding conformation of TNF with chrysophanol, chrysophanol is located within the channel formed by the TNF trimer. It forms hydrogen bonds with TYR119 in chain B, TYR151 in chain A, and TYR59 in chain A of TNF. It also forms hydrophobic interactions with multiple tyrosine residues in chains C and A of TNF, ensuring a stable binding interaction.

### 3.4 Active ingredients of LJZD inhibited U251 glioma cell viability, colony formation and trigger apoptosis

**FIGURE 8:**
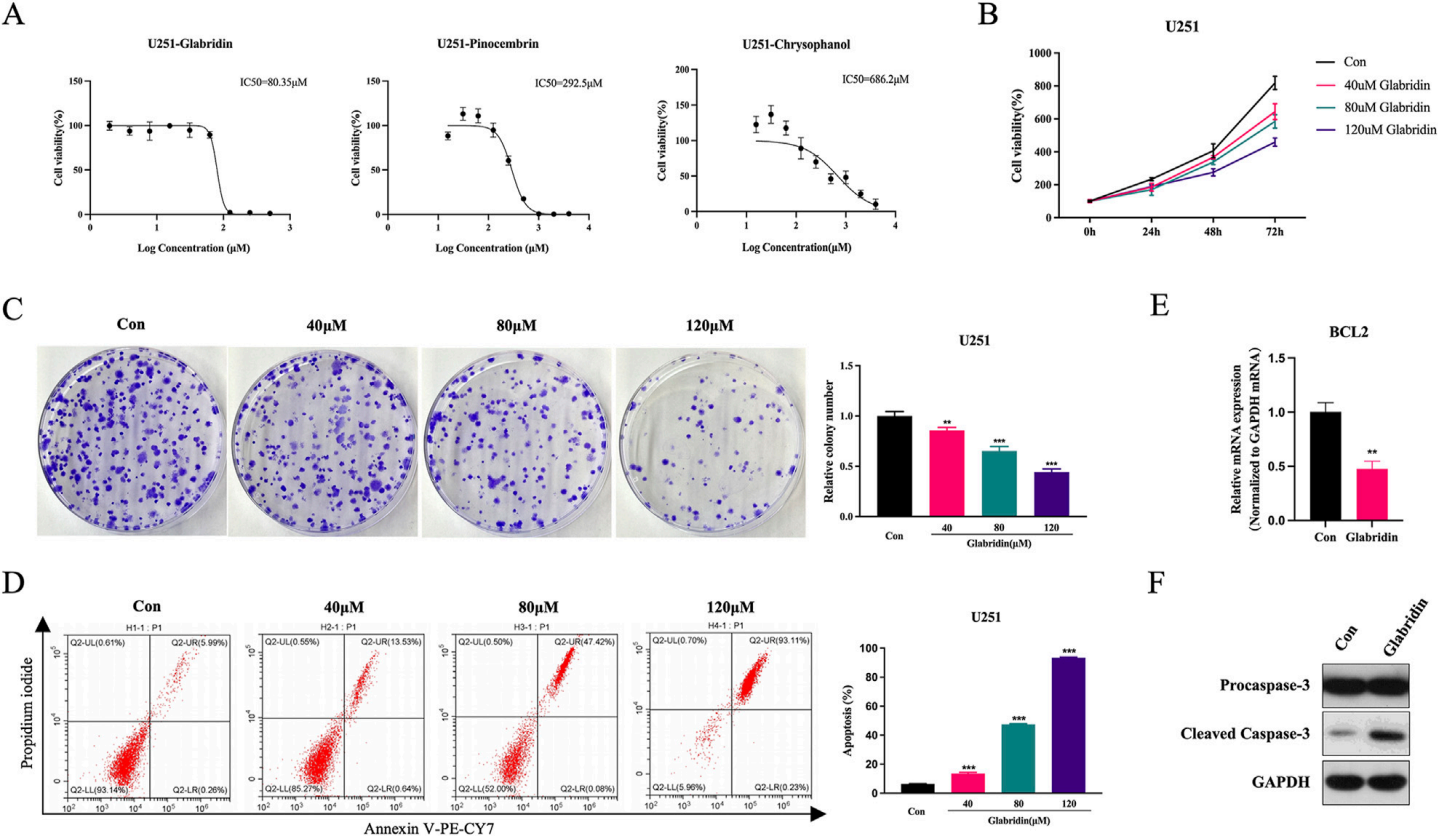
Inhibitory effects of active ingredients from LJZD on proliferation, clonogenicity and apoptosis induction in U251 glioma cells. **(A)** Determination of IC50 values for glabridin, pinocembrin and chrysophanol in U251 cells. The IC_50_ was defined as the compound concentration required to inhibit 50% of cell viability. Based on IC_50_ results, glabridin was selected for subsequent experiments at low (40 μM), medium (80 μM), and high (120 μM) concentrations. **(B)** Time- and dose-dependent effects of glabridin on U251 cell viability after 24, 48, and 72 h treatment. **(C)** Representative images and quantitative analysis of colony formation in U251 cells treated with various concentrations (40 μM, 80 μM, 120 μM) of glabridin. **(D)** Flow cytometry analysis and quantitative results showing glabridin-induced (40 μM, 80 μM, 120 μM) apoptosis in U251 cells. **(E)** Relative mRNA expression levels of BCL2 in U251 cells after treatment with 80 μM glabridin. **(F)** Western blot analysis demonstrating the effects of 80 μM glabridin on procaspase-3 and cleaved caspase-3 protein expression in U251 cells. All data are presented as mean ± SD. **P < 0.01; ***P < 0.001 vs. control group (Con).

As demonstrated by molecular docking analysis, glabridin, chrysophanol, and pinocembrin - exhibited strong binding affinity with the top 10 core targets (Supplementary Figure S1). Consequently, we selected these three compounds for further validation of their anti-glioma effects. Figure 8A shows that all three components significantly inhibited the viability of U251 glioma cells, with IC50 values of 80.35 μM for glabridin, 686.2 μM for chrysophanol, and 292.5 μM for pinocembrin. Given that glabridin not only demonstrated the lowest IC50 value but also showed the highest affinity with 8/10 of LJZD’s top anti-glioma core targets, we selected glabridin for subsequent experiments. Our results revealed that glabridin inhibited U251 cell viability in both concentration- and time-dependent manners (Figure 8B). Moreover, glabridin treatment markedly suppressed the colony-forming ability of U251 cells, with the inhibitory effect strengthening as concentrations increased (Figure 8C). Flow cytometry analysis further confirmed that glabridin effectively promoted apoptosis in U251 cells (Figure 8D). It is noteworthy that among LJZD’s core anti-glioma targets, BCL2 and CASP3 are key regulators of apoptosis. Elevated BCL2 expression enhances cellular resistance to apoptosis, while CASP3-encoded caspase-3 serves as the key executioner protease in the apoptotic cascade. Our findings demonstrated that treatment with 80 μM glabridin significantly downregulated BCL2 mRNA levels (Figure 8E) and markedly increased cleaved caspase-3 protein expression (Figure 8F) in U251 cells. These results collectively demonstrate that glabridin exerts anti-glioma effects by inducing apoptosis in glioma cells.

### 3.5 Active ingredients of LJZD modulates core targets related to drug resistance in glioma

**FIGURE 9:**
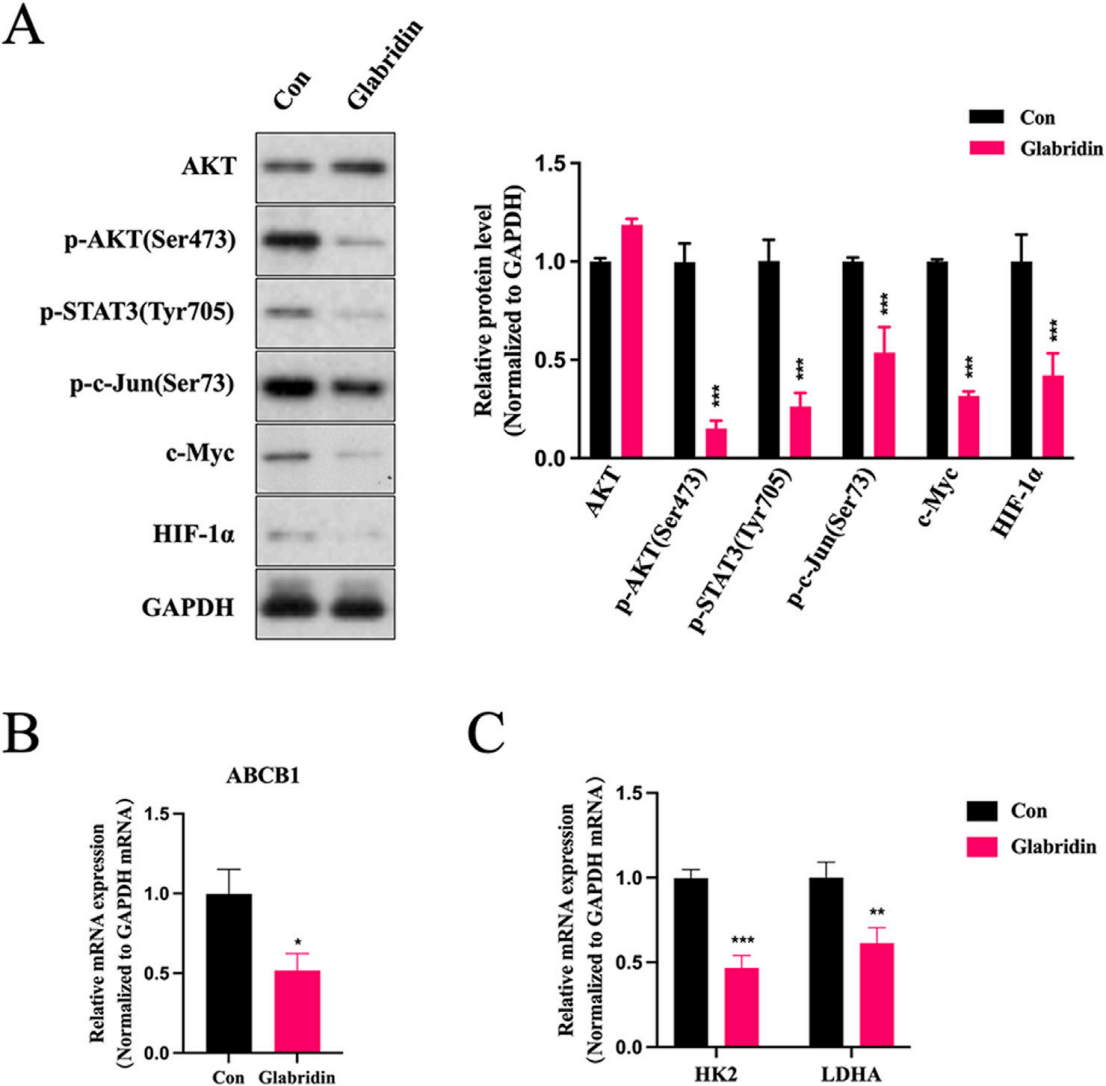
Effects of glabridin, an active ingredient of LJZD, on core targets and their downstream molecules in U251 cells. **(A)** Western blot analysis of total AKT, phosphorylated AKT (p-AKT), phosphorylated STAT3 (p-STAT3), phosphorylated c-Jun (p-c-Jun), c-Myc, and HIF-1α protein expression in U251 cells treated with glabridin (80 μM). GAPDH served as the internal control. The control group (Con) was treated with an equivalent volume of DMSO. Bar graphs represent densitometric quantification of protein levels. **(B,C)** RT-PCR analysis of mRNA expression levels of downstream targets ABCB1 **(B)** HK2 and LDHA **(C)**. GAPDH was used as the internal reference. *P < 0.05; **P < 0.01; ***P < 0.001 vs control group (Con).

To elucidate the molecular mechanisms by which glabridin modulates LJZD’s core anti-glioma targets, we systematically analyzed the expression changes of key signaling molecules in glabridin-treated U251 cells, including total AKT, p-AKT (Ser473), p-STAT3 (Tyr705), p-c-Jun (Ser73), as well as transcriptional regulators c-Myc and HIF-1α. Western blot analysis revealed (Figure 9A) that glabridin treatment significantly inhibited the phosphorylation activation of AKT, STAT3, and c-Jun, while reducing the protein expression levels of c-Myc and HIF-1α in U251 cells. KEGG pathway enrichment analysis demonstrated that LJZD’s core targets were significantly enriched in various signaling pathways related to drug resistance. Moreover, among these core targets, Akt, STAT3, and c-Jun have all been shown to transcriptionally regulate the expression of drug efflux pump ABCB1. Our results showed that glabridin treatment markedly downregulated ABCB1 mRNA expression in U251 cells (Figure 9B). Furthermore, based on the established roles of c-Myc and HIF-1α in regulating key glycolytic enzymes and promoting tumor drug resistance (Feng et al., 2020), we assessed the mRNA expression levels of HK2 and LDHA. The data demonstrated that glabridin treatment significantly suppressed the transcriptional activity of these two critical glycolytic enzymes (Figure 9C). These findings collectively demonstrate that glabridin, as one of the principal active ingredients of LJZD, can influence glioma drug resistance mechanisms through a multi-target regulatory network.

## 4 Discussion

Although there have been no previous reports on the application of LJZD in glioma treatment, it has demonstrated promising therapeutic effects in esophageal cancer (Han et al., 2023) and liver cancer (Guo et al., 2024). In this study, we identified 76 active ingredients from LJZD and obtained 174 intersection targets between LJZD and glioma. These intersection targets were enriched in 2164 BP terms, 243 MF terms, 149 CC terms, and 170 pathways. These results strongly suggest that LJZD may exert its anti-glioma effects through multiple active ingredients acting on multiple targets and pathways, which aligns with the multi-ingredient, multi-target, and multi-pathway characteristics of TCM((Chao et al., 2017)). The malignant proliferation, invasion, and treatment resistance of glioma are driven by a complex interplay of signaling pathways, including the p53, receptor tyrosine kinase, and Wnt signaling pathways, as well as cellular processes such as apoptosis, autophagy, senescence, and necrosis (Sottoriva et al., 2013; Patel et al., 2014). Given the intricate molecular network underlying these processes, multi-target and multi-pathway regulation is likely more effective than single-target drug therapy in controlling tumors, which is one of the advantages of TCM formulas. These formulas, composed of various herbs, can simultaneously modulate the same target or different nodes of the same pathway, thereby enhancing therapeutic efficacy through synergistic effects. As shown in Figure 5A, 71 out of 76 active ingredients in LJZD collectively influence 174 common targets, with targets such as PTGS2, ESR1, and NOS2 being regulated by over 30 active ingredients, highlighting the networked regulatory effects of LJZD on glioma-related targets and pathways.

PPI network analysis identified 40 key targets of LJZD in glioma, with TP53, AKT1, and HIF1A occupying central positions in the network topology. These targets exhibit both high degree and high betweenness, indicating that they serve as hubs for multiple pathways and critical control points for information flow within the network. They are also key molecules in the development, progression, and treatment resistance of glioma. It has been well established that the p53 pathway and the PI3K/Akt/mTOR pathway are central to glioblastoma tumorigenesis, pathophysiology, and resistance to treatment. Studies have shown that dysregulation of the p53-ARF-MDM2 pathway and PI3K/Akt pathway were observed in most glioma cases (Obrador et al., 2024; Cancer Genome Atlas Research, 2008). While disrupting p53-MDM2 interaction (Hao et al., 2023) or inhibiting PI3K/Akt pathway (Duchatel et al., 2024) effectively suppressed glioma progression *in vitro* and *in vivo*. HIF-1α, encoding by HIF1A, has been elucidated that its expression level is positively correlated with the pathological grade and microvascular density of glioma and negatively correlated with overall patient survival (Liu and Cao, 2015). Targeting HIF-1α is also one of the promising strategies for glioma treatment (Peng et al., 2021). Overall, given the pivotal roles of these targets in glioma, LJZD holds promise as a potential therapeutic strategy.

Our KEGG enrichment results revealed that apoptosis was the pathway with the highest fold enrichment score, while the GO analysis showed both “regulation of apoptotic process” and “regulation of programmed cell death” were among the top 10 BP terms, suggesting that inducing apoptosis is likely one of the mechanisms by which LJZD exerts its anti-glioma effects. *In vitro* experiments further demonstrated that all the three active ingredients (glabridin, chrysophanol, and pinocembrin) from LJZD significantly inhibited the viability of U251 glioma cells. While chrysophanol has previously been reported to suppress proliferation and induce apoptosis in U251 and SHG-44 cells (Gu et al., 2021), the effects of glabridin on glioma were unclear until the present study demonstrated that glabridin triggers apoptosis in U251 cells in a concentration-dependent manner, substantiating the pro-apoptotic activity of LJZD. Additionally, key targets involved in the core mechanisms of LJZD, such as TP53, BCL2, and CASP3, are closely related to apoptosis. Subsequent molecular docking further illuminated potential multi-target pro-apoptotic mechanisms. Small molecules that occupy the TP53-Y220C pocket stabilize the mutant protein and restore transcriptional activity (Baud et al., 2018), and six of the top ten LJZD constituents displayed binding energies below −7 kcal/mol to this pocket, with glabridin achieving the lowest at −8.2 kcal/mol, suggesting its potential to rescue mutant p53 function. The P2 hydrophobic pocket of BCL2 is the canonical inhibitory binding site (Liu et al., 2024), and both glabridin and chrysophanol docked here with favourable energies of −7.8 kcal/mol, implying neutralization of BCL2’s anti-apoptotic activity. Further validation revealed that glabridin exposure elevated cleaved-CASP3 and concurrently reduced BCL2 mRNA levels, consistent with previous reports of glabridin-mediated BCL2 repression in gastric (Liu et al., 2024) and breast cancers (Kodan et al., 2021), indicating a possible dual inhibition of BCL2 and concomitant apoptosis induction. Together, these findings position LJZD as a multi-ingredient, multi-target regimen that induces glioma apoptosis through stabilisation of mutant TP53, inhibition of BCL2 and activation of CASP3. Altogether, the convergence of network pharmacology, molecular docking and experimental validation frames LJZD as a multi-target pro-apoptotic regimen and underscores its translational promise for glioma therapy.

Among the top 10 KEGG pathways, several are associated with tumor pharmacotherapy, including platinum drug resistance and EGFR tyrosine kinase inhibitor resistance. Previous studies identified ABCB1-mediated efflux as a common mechanism underlying resistance to both platinum drugs and EGFR-TKIs (Kodan et al., 2021; Zhang et al., 2025; Hu et al., 2022). ABCB1 transcription is governed by PI3K/AKT, STAT3, HIF-1α and JNK pahways (Salaroglio et al., 2022; Schulz et al., 2023). We found that glabridin simultaneously reduced the protein levels of p-AKT, p-STAT3, HIF-1α and p-c-Jun in U251 cells, leading to a decrease in ABCB1 mRNA (Figure 9). These data suggest that glabridin may downregulates ABCB1 through convergent inhibition of multiple drug resistance-associated signaling axes. In parallel, glabridin suppressed the glycolytic enzymes HK2 and LDHA—two established HIF-1α targets that drive the Warburg effect. Consistent with reports that inhibition of Warburg effect sensitizes tumors to platinum agents and EGFR-TKIs (Tyagi et al., 2021; Dyrstad et al., 2021), our findings indicate that glabridin may augment drug efficacy via the HIF-1α–HK2/LDHA axis.

Temozolomide (TMZ) remains the first-line chemotherapeutic for glioblastoma, yet intrinsic or acquired resistance limits its efficacy. Although the present study did not directly examine TMZ, the glabridin-perturbed network we delineated aligns with a growing body of evidence linking each of these targets to TMZ sensitization. Studies show that blockade of LDHA ((Yue et al., 2024)) or HK2((Yang et al., 2024)) restores TMZ sensitivity in glioma cells, mirroring the reduced LDHA and HK2 expression we observed after glabridin treatment. Similarly, silencing HIF-1α increases glioma TMZ chemosensitivity (Wang et al., 2025), and STAT3 inhibition downregulates MGMT to overcome TMZ resistance (Cui et al., 2025), both of which are consistent with glabridin-mediated suppression of HIF-1α and p-STAT3. Furthermore, AKT/mTOR pathway inhibition enhances TMZ efficacy (Yin et al., 2024), paralleling the decreased p-AKT levels we report. Additionally, ABCB1 and related efflux transporters are established drivers of TMZ resistance, and their down-modulation restores TMZ sensitivity (Phon et al., 2025); reduction of ABCB1 expression by glabridin in U251 cells therefore positions it as a candidate for reversing this resistance mechanism. Collectively, these convergent literature findings suggest that the multi-pathway suppression effected by glabridin could meaningfully enhance TMZ responsiveness in glioma.

Finally, additional constituents of LJZD, such as isoliquiritigenin (Wang et al., 2019), shagaol (Hsu et al., 2023), and tangeretin (Xie et al., 2022), have independently been shown to restore TMZ, cisplatin or EGFR-TKIs sensitivity. Collectively, these observations position LJZD as a promising multi-ingredient regimen for overcoming chemo- and targeted-therapy resistance in glioma.

## 5 Conclusion

In conclusion, integrating network pharmacology with molecular docking and cellular assays, we delineated the multi-component, multi-target and multi-pathway mechanisms through which LJZD may counteract glioma. The study provided a cell-level findings for its use as an adjuvant sensitizer in combination with chemotherapy or targeted therapy, which should be validated fin animal models and clinical settings. Furthermore, in-depth exploration and necessary structural optimization of the core active ingredients screened in this study (such as pinocembrin, glabridin, or chrysophanol) may lead to the development of novel small-molecule drugs, thereby advancing clinical treatment progress for glioma.

## 6 Limitation

First, we applied Lipinski’s Rule of Five in combination with oral bioavailability and blood-brain barrier permeability to screen active ingredients in LJZD, aiming to prioritize candidate molecules with potential for oral drug development. However, this approach may exclude certain pharmacologically active components, such as polysaccharides and saponins, due to their molecular weight or polarity exceeding the predefined criteria. Although some active ingredients demonstrate suboptimal oral absorption or blood-brain barrier penetration, their therapeutic efficacy could potentially be enhanced through functional group modifications or alternative administration routes (e.g., intravenous injection or localized delivery). Therefore, the therapeutic potential of other active ingredients excluded by our screening criteria should not be disregarded. Second, to elucidate LJZD’s effects on core targets, we selected glabridin for experimental validation based on molecular docking results, which yielded promising results. It should be noted that glabridin was identified from 76 active ingredients in LJZD, and other ingredients may also possess considerable anti-glioma activity warranting further investigation. Meanwhile, further identification of LJZD active ingredients by mass-spectrometric analysis may yield additional novel findings. Third, the mechanistic validation reported here was conducted primarily in U251 cells; future work should extend these observations to additional glioma cell lines, primary cultures, and *in vivo* models. Moreover, the synergistic interactions among multiple ingredients represent a crucial aspect of TCM’s multi-ingredient, multi-target mechanism. Building upon single-component studies, exploring the network-based regulatory effects of multiple ingredients on key targets and pathways will provide a more comprehensive understanding of LJZD’s anti-glioma mechanisms and advance glioma treatment development.

## Data Availability

The raw data supporting the conclusions of this article will be made available by the authors, without undue reservation.
