# Supplementary material for: Multi-pathway mechanisms of Liujunzi decoction in promoting glioma apoptosis and reversing drug resistance: network pharmacology and experimental validation
Source: Front Pharmacol. 2025 Sep 24;16:1682550. doi: 10.3389/fphar.2025.1682550 (PMC12504358; doi:10.3389/fphar.2025.1682550)
Supplement: Supplementary file 1 [file Table1.docx]

Supplementary Material

# Supplementary Figures and Tables

## Supplementary Tables

| **Supplementary Table 1.** Primer sequences for RT-PCR. | | |
| --- | --- | --- |
| Gene Name | Primer sequences | Size (bp) |
| Bcl-2-F | GGTGGGGTCATGTGTGTGG | 89 |
| Bcl-2-R | CGGTTCAGGTACTCAGTCATCC |  |
| ABCB1-F | GGGATGGTCAGTGTTGATGGA | 110 |
| ABCB1-R | GCTATCGTGGTGGCAAACAATA |  |
| HK2-F | TTGACCAGGAGATTGACATGGG | 248 |
| HK2-R | CAACCGCATCAGGACCTCA |  |
| LDHA-F | ATGGCAACTCTAAAGGATCAGC | 86 |
| LDHA-R | CCAACCCCAACAACTGTAATCT |  |
| GAPDH-F | GGAGCGAGATCCCTCCAAAT | 197 |
| GAPDH-R | GGCTGTTGTCATACTTCTCATGG |  |

| **Supplementary Table 2.** Active ingredients of Liujunzi decoction (LJZD). | | | | |
| --- | --- | --- | --- | --- |
| Code | Parent Herb | Ingredient Name | PubChem CID | InChIKey |
| RS1 | RS | Mycosinol | 5281153 | FZRGCIPZQGXDCM-IZZDOVSWSA-N |
| RS2 | RS | Aponorhyoscine | 5319581 | UPWMWFSEBOFTNA-UHFFFAOYSA-N |
| RS3 | RS | Elemicin | 10248 | BPLQKQKXWHCZSS-UHFFFAOYSA-N |
| RS4 | RS | Protopine | 4970 | GPTFURBXHJWNHR-UHFFFAOYSA-N |
| RS5 | RS | Frutinone A | 441965 | RFWULRHBGYKEEZ-UHFFFAOYSA-N |
| RS6 | RS | Cadin-4-en-10-ol | 10398656 | LHYHMMRYTDARSZ-BYNSBNAKSA-N |
| RS7 | RS | Panaxytriol | 93484 | RDIMTXDFGHNINN-UHFFFAOYSA-N |
| RS8 | RS | Di-tert-butyl phthalate | 121712 | RYCNBIYTZSGSPI-UHFFFAOYSA-N |
| RS9 | RS | alpha-Humulene epoxide | 14038843 | RKQDKXOBRXTSFS-UOAUIWSESA-N |
| RS10 | RS | Ginsenoyne A | 5317632 | FTXZFRIHQNXZNH-UHFFFAOYSA-N |
| RS11 | RS | alpha-Farnesene, (3Z,6Z)- | 5317320 | CXENHBSYCFFKJS-LOQWIJHWSA-N |
| RS12 | RS | (-)-Maalioxide | 12085452 | PZKNYJWHOZUWDF-MYZSUADSSA-N |
| RS13 | RS | Ginsenoyne B | 5317633 | MORPELUWUARUFU-UHFFFAOYSA-N |
| RS14 | RS | Girinimbine | 96943 | GAEQWKVGMHUUKO-UHFFFAOYSA-N |
| RS15 | RS | Beta-Santalol | 6857681 | OJYKYCDSGQGTRJ-GQYWAMEOSA-N |
| BZ1 | BZ | Furanodiene | 9601230 | VMDXHYHOJPKFEK-RZWYOFBOSA-N |
| BZ2 | BZ | Selina-4(15),7(11)-dien-8-one | 13986100 | NKGSEACIYQINQJ-DZGCQCFKSA-N |
| BZ3 | BZ | Elemol | 92138 | GFJIQNADMLPFOW-VNHYZAJKSA-N |
| BZ4 | BZ | beta-EUDESMOL | 91457 | BOPIMTNSYWYZOC-VNHYZAJKSA-N |
| BZ5 | BZ | 1-Naphthalenol, decahydro-1,4a-dimethyl-7-(1-methylethylidene)- | 521214 | STRABSCAWZINIF-UHFFFAOYSA-N |
| BZ6 | BZ | Hinesol | 10878761 | ICWHTQRTTHCUHW-GZBFAFLISA-N |
| BZ7 | BZ | Atractylenolide I | 5321018 | ZTVSGQPHMUYCRS-SWLSCSKDSA-N |
| **Supplementary Table 2.** Active ingredients of Liujunzi decoction (LJZD). (*continued*). | | | | |
| BZ8 | BZ | (4aR,8aS,9aR)-3,8a-dimethyl-5-methylidene-4a,6,7,8,9,9a-hexahydro-4H-benzo[f][1]benzofuran-2-one | 11736433 | OQYBLUDOOFOBPO-NFAWXSAZSA-N |
| BZ9 | BZ | Atractylenolide Iii | 155948 | FBMORZZOJSDNRQ-GLQYFDAESA-N |
| BZ10 | BZ | (4aS,8aR,9aS)-9a-Ethoxy-3,8a-dimethyl-5-methylene-4a,5,6,7,8,8a,9,9a-octahydronaphtho[2,3-b]furan-2(4H)-one | 14448075 | JATCNILBQFTWFJ-XKQJLSEDSA-N |
| BZ11 | BZ | [(3R,4E,6Z,12E)-1-acetyloxy-14-hydroxytetradeca-4,6,12-trien-8,10-diyn-3-yl] 3-methylbut-2-enoate | 132941086 | CQDVFBMTEZFKKY-PUYRJQRLSA-N |
| CP1 | CP | Diethyl Phthalate | 6781 | FLKPEMZONWLCSK-UHFFFAOYSA-N |
| CP2 | CP | Tangeretin | 68077 | ULSUXBXHSYSGDT-UHFFFAOYSA-N |
| GC1 | GC | alpha-Cadinol | 519662 | LHYHMMRYTDARSZ-UHFFFAOYSA-N |
| GC2 | GC | Dibenzoylmethane | 8433 | NZZIMKJIVMHWJC-UHFFFAOYSA-N |
| GC3 | GC | (Z)-1-(2,4-dihydroxyphenyl)-3-phenylprop-2-en-1-one | 10331849 | JUMSUVHHUVPSOY-TWGQIWQCSA-N |
| GC4 | GC | 7-hydroxy-2-methyl-3-phenyl-4H-chromen-4-one | 5380976 | BBCDTCKKROIGAB-UHFFFAOYSA-N |
| GC5 | GC | 4',7-Dihydroxyflavone | 5282073 | LCAWNFIFMLXZPQ-UHFFFAOYSA-N |
| GC6 | GC | Liquiritigenin | 114829 | FURUXTVZLHCCNA-AWEZNQCLSA-N |
| GC7 | GC | Pinocembrin | 68071 | URFCJEUYXNAHFI-ZDUSSCGKSA-N |
| GC8 | GC | Trihydroxychalcone | 638278 | DXDRHHKMWQZJHT-FPYGCLRLSA-N |
| GC9 | GC | 7-Methoxy-2-methyl-3-phenyl-4H-chromen-4-one | 354368 | XRGWZIGGCNSFRY-UHFFFAOYSA-N |
| GC10 | GC | Formononetin | 5280378 | HKQYGTCOTHHOMP-UHFFFAOYSA-N |
| GC11 | GC | Isoformononetin | 3764 | LNIQZRIHAMVRJA-UHFFFAOYSA-N |
| GC12 | GC | 3-(2-hydroxy-4-methoxyphenyl)-2H-chromen-7-ol | 10378419 | CXCORJXYCPSBSK-UHFFFAOYSA-N |
| GC13 | GC | Medicarpin | 336327 | NSRJSISNDPOJOP-BBRMVZONSA-N |
| GC14 | GC | Echinatin | 6442675 | QJKMIJNRNRLQSS-WEVVVXLNSA-N |
| **Supplementary Table 2.** Active ingredients of Liujunzi decoction (LJZD). (*continued*). | | | | |
| GC15 | GC | (+)-Vestitol | 177149 | XRVFNNUXNVWYTI-LLVKDONJSA-N |
| GC16 | GC | 4',7-Dimethoxyisoflavone | 136419 | LPNBCGIVZXHHHO-UHFFFAOYSA-N |
| GC17 | GC | Glypallichalcone | 5317768 | CTBBBAQHUJVKNG-UXBLZVDNSA-N |
| GC18 | GC | 7-Acetoxy-2-methylisoflavone | 268208 | DPIAJERHFDBLPT-UHFFFAOYSA-N |
| GC19 | GC | Afrormosin | 5281704 | KJGPBYUQZLUKLL-UHFFFAOYSA-N |
| GC20 | GC | Glabrocoumarone A | 10542808 | SJIZTMNAKZAOTA-UHFFFAOYSA-N |
| GC21 | GC | Shinpterocarpin | 10336244 | QGPHRCQDTPCIQI-KXBFYZLASA-N |
| GC22 | GC | Glabrene | 480774 | NGGYSPUAKQMTNP-UHFFFAOYSA-N |
| GC23 | GC | Inflacoumarin A | 5318437 | RNBLSJGPSGNSIN-UHFFFAOYSA-N |
| GC24 | GC | Kanzonol B | 10881804 | TUHJQMZJOMZXJO-XVNBXDOJSA-N |
| GC25 | GC | 1-(5-Hydroxy-2,2-dimethylchromen-6-yl)-3-(4-hydroxyphenyl)prop-2-en-1-one | 3662729 | IQHPDUUSMBMDGN-UHFFFAOYSA-N |
| GC26 | GC | Phaseollinisoflavan | 162412 | UUJBHSNXZMGYBT-ZDUSSCGKSA-N |
| GC27 | GC | Glabridin | 124052 | LBQIJVLKGVZRIW-ZDUSSCGKSA-N |
| GC28 | GC | Isobavachin | 193679 | KYFBXCHUXFKMGQ-IBGZPJMESA-N |
| GC29 | GC | Glabranin | 124049 | DAWSYIQAGQMLFS-SFHVURJKSA-N |
| GC30 | GC | 4'-O-Methylglabridin | 9927807 | ZZAIPFIGEGQNHP-AWEZNQCLSA-N |
| GC31 | GC | Licoagrocarpin | 15840593 | NYWUHXBEDBSRQB-UWJYYQICSA-N |
| GC32 | GC | Licochalcone C | 9840805 | WBDNTJSRHDSPSR-KPKJPENVSA-N |
| GC33 | GC | Licochalcone A | 5318998 | KAZSKMJFUPEHHW-DHZHZOJOSA-N |
| GC34 | GC | (3S)-3-[2-hydroxy-4-methoxy-3-(3-methylbut-2-enyl)phenyl]-3,4-dihydro-2H-chromen-7-ol | 162845229 | RMIHCTUWJGVJQB-OAHLLOKOSA-N |
| GC35 | GC | 3 inverted exclamation marka-Hydroxy-4 inverted exclamation marka-O-methylglabridin | 15228662 | PPBISUGOQDBBEL-ZDUSSCGKSA-N |
| GC36 | GC | 3'-Methoxyglabridin | 5319439 | SBQBKTSYEKPBJF-UHFFFAOYSA-N |
| **Supplementary Table 2.** Active ingredients of Liujunzi decoction (LJZD). (*continued*). | | | | |
| GC37 | GC | 1-Methoxyphaseollidin | 480873 | YKTZRMXYANFKQR-YCRPNKLZSA-N |
| GC38 | GC | Glyasperin D | 480860 | DDMAUIOCNQXFHL-AWEZNQCLSA-N |
| GC39 | GC | Xambioona | 14769500 | FGJUXFVUOCKRCY-UHFFFAOYSA-N |
| BX1 | BX | Cedrol | 65575 | SVURIXNDRWRAFU-OGMFBOKVSA-N |
| BX2 | BX | Chrysophanic acid | 10208 | LQGUBLBATBMXHT-UHFFFAOYSA-N |
| BX3 | BX | Shogaol | 5281794 | OQWKEEOHDMUXEO-BQYQJAHWSA-N |
| BX4 | BX | Neocryptotanshinone | 389888 | LGZFJHSOBYVDLA-JTQLQIEISA-N |
| BX5 | BX | Cavidine | 193148 | JTZZGWPIBBTYNE-FKIZINRSSA-N |
| A | RS, GC | Maackiain | 91510 | HUKSJTUUSUGIDC-ZBEGNZNMSA-N |
| B | RS, GC | Dibutyl Phthalate | 3026 | DOIRQSBPFJWKBE-UHFFFAOYSA-N |
| C | BZ, CP, GC | Diisobutyl phthalate | 6782 | MGWAVDBGNNKXQV-UHFFFAOYSA-N |
| D | FL, CP | Lauric Acid | 3893 | POULHZVOKOAJMA-UHFFFAOYSA-N |
| RS Panax ginseng C. A. Mey., BZ Atractylodes macrocephala Koidz., FL Poria cocos (Schw.) Wolf., GC licorice, BX Pinellia ternata (Thunb.), ChP Citrus reticulata Blanco. | | | | |

| **Supplementary Table 3.** GO term analysis of intersection targets between LJZD and glioma. | | | | | | | | |
| --- | --- | --- | --- | --- | --- | --- | --- | --- |
| GO Term ID | Term | Category | Count | % | Fold Enrichment | Benjamini | FDR |  |
| 19899 | enzyme binding | MF | 88 | 50.57471264 | 4.538797086 | 9.62E-35 | 7.57E-35 |  |
| 42802 | identical protein binding | MF | 80 | 45.97701149 | 3.913414532 | 2.09E-26 | 1.64E-26 |  |
| 8134 | transcription factor binding | MF | 39 | 22.4137931 | 7.131424327 | 4.00E-19 | 3.15E-19 |  |
| 61629 | RNA polymerase II-specific DNA-binding transcription factor binding | MF | 30 | 17.24137931 | 9.249518304 | 3.61E-17 | 2.84E-17 |  |
| 140297 | DNA-binding transcription factor binding | MF | 34 | 19.54022989 | 7.517536789 | 3.61E-17 | 2.84E-17 |  |
| 44389 | ubiquitin-like protein ligase binding | MF | 28 | 16.09195402 | 9.592093056 | 2.05E-16 | 1.61E-16 |  |
| 31625 | ubiquitin protein ligase binding | MF | 27 | 15.51724138 | 9.858039246 | 4.25E-16 | 3.34E-16 |  |
| 19900 | kinase binding | MF | 40 | 22.98850575 | 5.44089312 | 4.99E-16 | 3.93E-16 |  |
| 19904 | protein domain specific binding | MF | 36 | 20.68965517 | 6.109773559 | 9.37E-16 | 7.37E-16 |  |
| 19901 | protein kinase binding | MF | 37 | 21.26436782 | 5.579872455 | 4.63E-15 | 3.64E-15 |  |
| 5829 | cytosol | CC | 113 | 64.94252874 | 2.344146111 | 9.78E-22 | 7.51E-22 |  |
| 32991 | protein-containing complex | CC | 112 | 64.36781609 | 2.025879912 | 2.07E-16 | 1.59E-16 |  |
| 5737 | cytoplasm | CC | 158 | 90.8045977 | 1.475481013 | 2.07E-16 | 1.59E-16 |  |
| 43233 | organelle lumen | CC | 103 | 59.1954023 | 1.918350694 | 1.44E-12 | 1.11E-12 |  |
| 31974 | membrane-enclosed lumen | CC | 103 | 59.1954023 | 1.918350694 | 1.44E-12 | 1.11E-12 |  |
| 70013 | intracellular organelle lumen | CC | 103 | 59.1954023 | 1.918350694 | 1.44E-12 | 1.11E-12 |  |
| 31982 | vesicle | CC | 80 | 45.97701149 | 2.233237532 | 7.26E-12 | 5.58E-12 |  |
| 31410 | cytoplasmic vesicle | CC | 60 | 34.48275862 | 2.741139554 | 1.22E-11 | 9.37E-12 |  |
| 97708 | intracellular vesicle | CC | 60 | 34.48275862 | 2.731773474 | 1.26E-11 | 9.67E-12 |  |
| 5739 | mitochondrion | CC | 50 | 28.73563218 | 3.105252602 | 2.89E-11 | 2.22E-11 |  |
| 70887 | cellular response to chemical stimulus | BP | 127 | 72.98850575 | 6.899014599 | 5.60E-81 | 3.99E-81 |  |
| 42221 | response to chemical | BP | 150 | 86.20689655 | 4.365840789 | 1.14E-76 | 8.13E-77 |  |
| 1901700 | response to oxygen-containing compound | BP | 113 | 64.94252874 | 7.562389486 | 3.69E-73 | 2.63E-73 |  |
| 43067 | regulation of programmed cell death | BP | 109 | 62.64367816 | 7.373841056 | 1.40E-68 | 9.97E-69 |  |
| 42981 | regulation of apoptotic process | BP | 107 | 61.49425287 | 7.449590499 | 2.00E-67 | 1.43E-67 |  |
| **Supplementary Table 3.** GO term analysis of intersection targets between LJZD and glioma. (*continued*). | | | | | | | |  |
| 1901701 | cellular response to oxygen-containing compound | BP | 91 | 52.29885057 | 8.888633997 | 3.09E-61 | 2.21E-61 |  |
| 9628 | response to abiotic stimulus | BP | 91 | 52.29885057 | 8.662460104 | 2.59E-60 | 1.84E-60 |  |
| 6950 | response to stress | BP | 134 | 77.01149425 | 4.076709856 | 3.04E-59 | 2.16E-59 |  |
| 9719 | response to endogenous stimulus | BP | 90 | 51.72413793 | 7.793834297 | 1.32E-55 | 9.44E-56 |  |
| 51716 | cellular response to stimulus | BP | 160 | 91.95402299 | 2.688575269 | 1.87E-55 | 1.33E-55 |  |
| MF molecular function, CC cellular component, BP biological process. | | | | | | | | |

| **Supplementary Table 4.** The top 50 enriched KEGG pathways derived from analysis of intersection targets between LJZD and glioma. | | | | | | |
| --- | --- | --- | --- | --- | --- | --- |
| KEGG ID | Term | Count | % | Fold Enrichment | Benjamini | FDR |
| hsa04215 | Apoptosis - multiple species | 11 | 6.32183908 | 17.35835799 | 1.03E-09 | 4.01E-10 |
| hsa05219 | Bladder cancer | 14 | 8.045977011 | 17.24289219 | 2.05E-12 | 7.96E-13 |
| hsa01524 | Platinum drug resistance | 25 | 14.36781609 | 16.83234714 | 6.32E-22 | 2.46E-22 |
| hsa04933 | AGE-RAGE signaling pathway in diabetic complications | 33 | 18.96551724 | 16.49903334 | 5.56E-29 | 2.16E-29 |
| hsa05134 | Legionellosis | 18 | 10.34482759 | 16.23119189 | 1.53E-15 | 5.94E-16 |
| hsa01521 | EGFR tyrosine kinase inhibitor resistance | 24 | 13.79310345 | 15.14911243 | 4.64E-20 | 1.80E-20 |
| hsa05212 | Pancreatic cancer | 23 | 13.2183908 | 15.08353185 | 3.58E-19 | 1.39E-19 |
| hsa05133 | Pertussis | 22 | 12.64367816 | 14.24275527 | 8.39E-18 | 3.26E-18 |
| hsa04917 | Prolactin signaling pathway | 20 | 11.49425287 | 14.22451871 | 3.57E-16 | 1.39E-16 |
| hsa05210 | Colorectal cancer | 24 | 13.79310345 | 13.93021832 | 3.50E-19 | 1.36E-19 |
| hsa01522 | Endocrine resistance | 27 | 15.51724138 | 13.77192039 | 1.94E-21 | 7.52E-22 |
| hsa05220 | Chronic myeloid leukemia | 21 | 12.06896552 | 13.77192039 | 1.06E-16 | 4.13E-17 |
| hsa05142 | Chagas disease | 28 | 16.09195402 | 13.72735107 | 4.17E-22 | 1.62E-22 |
| hsa05215 | Prostate cancer | 26 | 14.94252874 | 13.39717425 | 2.37E-20 | 9.20E-21 |
| hsa05417 | Lipid and atherosclerosis | 55 | 31.6091954 | 12.85804295 | 3.29E-44 | 1.28E-44 |
| hsa05213 | Endometrial cancer | 15 | 8.620689655 | 12.83823087 | 1.98E-11 | 7.69E-12 |
| hsa04370 | VEGF signaling pathway | 15 | 8.620689655 | 12.62426036 | 2.46E-11 | 9.57E-12 |
| hsa05222 | Small cell lung cancer | 23 | 13.2183908 | 12.48851562 | 2.28E-17 | 8.87E-18 |
| hsa04066 | HIF-1 signaling pathway | 27 | 15.51724138 | 12.39472835 | 2.66E-20 | 1.03E-20 |
| hsa05235 | PD-L1 expression and PD-1 checkpoint pathway in cancer | 22 | 12.64367816 | 12.34372124 | 1.69E-16 | 6.56E-17 |
| hsa05140 | Leishmaniasis | 19 | 10.91954023 | 12.14485806 | 4.45E-14 | 1.73E-14 |
| hsa04115 | p53 signaling pathway | 18 | 10.34482759 | 12.11928994 | 2.39E-13 | 9.29E-14 |
| hsa05230 | Central carbon metabolism in cancer | 17 | 9.770114943 | 12.0908409 | 1.44E-12 | 5.60E-13 |
| hsa05161 | Hepatitis B | 39 | 22.4137931 | 12.0821142 | 4.74E-29 | 1.84E-29 |
| hsa04210 | Apoptosis | 32 | 18.3908046 | 11.8816568 | 2.13E-23 | 8.27E-24 |
| hsa05418 | Fluid shear stress and atherosclerosis | 33 | 18.96551724 | 11.8184565 | 5.53E-24 | 2.15E-24 |
| hsa01523 | Antifolate resistance | 7 | 4.022988506 | 11.782643 | 4.83E-05 | 2.14E-05 |
| hsa04657 | IL-17 signaling pathway | 22 | 12.64367816 | 11.6940517 | 5.13E-16 | 1.99E-16 |
| hsa05162 | Measles | 32 | 18.3908046 | 11.62521817 | 3.81E-23 | 1.48E-23 |
| hsa04931 | Insulin resistance | 25 | 14.36781609 | 11.58189023 | 4.51E-18 | 1.75E-18 |
| hsa04012 | ErbB signaling pathway | 19 | 10.91954023 | 11.1563231 | 1.88E-13 | 7.30E-14 |
| hsa05221 | Acute myeloid leukemia | 15 | 8.620689655 | 11.13905325 | 1.40E-10 | 5.45E-11 |
| hsa05218 | Melanoma | 16 | 9.195402299 | 11.06784469 | 3.00E-11 | 1.17E-11 |
| hsa05216 | Thyroid cancer | 8 | 4.597701149 | 10.91827923 | 1.49E-05 | 6.33E-06 |
| **Supplementary Table 4.** The top 50 enriched KEGG pathways derived from analysis of intersection targets between LJZD and glioma. (*continued*). | | | | | | |
| hsa05143 | African trypanosomiasis | 8 | 4.597701149 | 10.91827923 | 1.49E-05 | 6.33E-06 |
| hsa04920 | Adipocytokine signaling pathway | 15 | 8.620689655 | 10.82079459 | 2.10E-10 | 8.15E-11 |
| hsa04930 | Type II diabetes mellitus | 10 | 5.747126437 | 10.74405137 | 6.68E-07 | 2.59E-07 |
| hsa04668 | TNF signaling pathway | 25 | 14.36781609 | 10.60862215 | 3.12E-17 | 1.21E-17 |
| hsa04213 | Longevity regulating pathway - multiple species | 13 | 7.471264368 | 10.58808933 | 6.80E-09 | 2.64E-09 |
| hsa04625 | C-type lectin receptor signaling pathway | 22 | 12.64367816 | 10.58033249 | 3.97E-15 | 1.54E-15 |
| hsa05223 | Non-small cell lung cancer | 15 | 8.620689655 | 10.3761044 | 3.72E-10 | 1.44E-10 |
| hsa04932 | Non-alcoholic fatty liver disease | 32 | 18.3908046 | 10.29239061 | 1.28E-21 | 4.99E-22 |
| hsa05144 | Malaria | 10 | 5.747126437 | 10.09940828 | 1.15E-06 | 4.49E-07 |
| hsa05214 | Glioma | 15 | 8.620689655 | 9.966521333 | 6.48E-10 | 2.52E-10 |
| hsa05145 | Toxoplasmosis | 22 | 12.64367816 | 9.919061708 | 1.68E-14 | 6.51E-15 |
| hsa05135 | Yersinia infection | 27 | 15.51724138 | 9.87985593 | 8.05E-18 | 3.13E-18 |
| hsa04068 | FoxO signaling pathway | 26 | 14.94252874 | 9.871602082 | 3.45E-17 | 1.34E-17 |
| hsa04620 | Toll-like receptor signaling pathway | 21 | 12.06896552 | 9.728787797 | 9.85E-14 | 3.82E-14 |
| hsa04659 | Th17 cell differentiation | 21 | 12.06896552 | 9.728787797 | 9.85E-14 | 3.82E-14 |
| hsa04926 | Relaxin signaling pathway | 25 | 14.36781609 | 9.710969504 | 2.40E-16 | 9.33E-17 |
